# Small Intestinal Bacterial Overgrowth: Comprehensive Review of Diagnosis, Prevention, and Treatment Methods

**DOI:** 10.7759/cureus.8860

**Published:** 2020-06-27

**Authors:** Ted George O Achufusi, Anuj Sharma, Ernesto A Zamora, Divey Manocha

**Affiliations:** 1 Internal Medicine, State University of New York Upstate Medical University, Syracuse, USA; 2 Gastroenterology, State University of New York Upstate Medical University, Syracuse, USA

**Keywords:** diarrhea, dysmotility, hydrogen breath testing, inflammatory bowel disease, malabsorption, microbiome, rifaxamin, small intestinal bacterial overgrowth

## Abstract

Small intestinal bacterial overgrowth (SIBO) is a commonly diagnosed gastrointestinal disorder affecting millions of individuals throughout the United States. It refers to a condition in which there is an excess and imbalance of small intestinal bacteria. Despite its prevalence, it remains underdiagnosed due to the invasive nature of diagnostic testing. Symptoms observed in SIBO, including abdominal distension, bloating, diarrhea, and gas formation, are nonspecific and can overlap with other gastrointestinal disorders. Frequently cited predisposing factors include gastric acid suppression, dysmotility, gastric bypass, and opioids. The diagnostic gold standard remains small bowel aspirate and culture. However, due to its invasive nature, it remains an unpopular method among patients and clinicians alike. Glucose and lactulose breath testing have become the go-to diagnostic method in clinical practice due to its noninvasive nature and low cost. Treatment is guided towards the eradication of bacteria in the small bowel and usually consists of a prolonged course of oral antibiotics. Due to recent advances in our understanding of the human microbiome, we are surely poised for a transformation in our approach to diagnosing and treating this condition.

## Introduction and background

Small intestinal bacterial overgrowth (SIBO) is a well-recognized cause of maldigestion and malabsorption worldwide. Historically, SIBO was widely considered a controversial and contested diagnosis. However, it has recently gained traction as a viable explanation for a wide variety of gastrointestinal manifestations. Much of the controversy surrounding the diagnosis stems from the wide-ranging clinical presentations and substantial overlap with other heterogeneous diagnoses, with irritable bowel syndrome (IBS) being the most cited example. The ambiguity surrounding SIBO is compounded by the lack of consensus when it comes to diagnosing and treating the condition. However, much has changed recently in our understanding of SIBO and how to approach treatment. Previously used jejunal aspirates and duodenal fluid analyses have been replaced by breath tests, while newly published guidelines provide a clearer picture of how to interpret diagnostic data, such as breath testing. In this comprehensive review, we will provide a current and up-to-date review of small intestinal bacterial overgrowth, including prevalence, symptomology, predisposing risk factors, the evolution of diagnostic testing, and the latest in available treatment options while focusing on their interface with diet and nutrition. 

## Review

Normal human microbiome

Gaining a better understanding of the small intestinal microbiome has proven to be key in unraveling SIBO, as it helps clinicians determine the validity of any diagnostic method used to screen for this condition. The current understanding of the human microbiome has progressed rapidly over recent years. However, one can say we are still at the infancy stages, as much has to be learned about the microbiome outside of the colon. Historically, most human studies of the gut microbiome have been based on fecal studies that provide valuable information on the large intestine microbiome, but they fall short in answering key questions regarding bacteria inhabiting the small intestine.

The human gut is inhabited by 10^14^ bacterial cells, which is roughly 10 times higher than the number of cells in the human body [1]. This diverse microbiome is composed of a wide range of organisms, including bacteria, fungi, and viruses. Bacteria compromise the largest portion of this microbiome, with approximately 500 to 1,000 different bacterial species identified to date [2]. The number of bacteria increases with progression from the proximal small intestine to the large intestine. The small intestine is comprised of mainly gram-positive and aerobic bacteria, while the large intestine contains predominantly gram-negative and anaerobic bacteria. The major phyla comprising the gut includes *Bacteroidetes* and *Firmicutes,* whilst *Actinobacteria, Fusobacteria, Verrucomicrobia,* and *Cyanobacteria* are also present, albeit in a smaller proportion [3].

For known organisms, culturing remains the most sensitive detection method, allowing for classification of isolate based on antibiotic resistance, antibiotic resistance mechanisms, and pathogenicity. However, this method is most suitable for the detection of a small number of well-known aerobic organisms and does not allow for recognition of the large complex anaerobic gut microbiome. Over the past four years, analysis of the gut microbiome has become more practical due to remarkable advances in next-generation sequencing (NGS) technologies, which enable researchers to comprehensively analyze the entire human microbiome community structures, including difficult-to-culture microbes [4].

Risk factors

The prevalence of SIBO among the general population is unknown. However, in most studies, SIBO has been detected anywhere from 0 to 20% of healthy controls [5]. The most common risk factors for abnormal or excessive small bowel bacterial overgrowth include disturbances in the small bowel anatomy and motility. Frequently cited examples include diabetic enteropathy, underlying connective tissue disease, chronic opiate use, diverticula, small bowel adhesions, and blind limbs. Additionally, impairments in the normal biochemical clearance of bacteria also predispose to bacterial overgrowth. This includes hypochloremia caused by chronic proton pump inhibitor (PPI) use and reduced pancreaticobiliary secretions caused by chronic pancreatitis.

Dysmotility has long been documented as a potential risk factor for bacterial overgrowth. A recent study (n = 150) designed to investigate the role of dysmotility and PPI use in patients with persistent gastrointestinal complaints demonstrated that patients with small intestinal dysmotility have an increased risk for SIBO based on duodenal aspirate/culture (>10^3^ colony-forming units (CFU)/mL threshold, odds ratio (OR) 3.6; P = 0.0003) [6]. Other gastrointestinal disorders have also been linked with the development of SIBO. Most notably, inflammatory bowel disease (IBD), dyspepsia, pancreatitis, and history of prior colectomy have all been cited as potential risk factors for bacterial overgrowth. In a 2018 case-control study, patients who underwent colectomy were diagnosed with SIBO at a much higher percentage compared to those with longstanding gastrointestinal symptoms without a prior colectomy (62% vs 32%, respectively, P = 0.0005) [7].

A 2019 meta-analysis conducted with the aim to review the prevalence of SIBO among patients with ulcerative colitis and Crohn’s disease showed a direct correlation between IBD and SIBO [8]. There were 11 studies included as part of the meta-analysis with combined 1,175 adult patients with IBD and 407 controls. Breath testing was utilized for SIBO diagnosis in each of the 11 studies. The prevalence of SIBO in IBD patients was 22.3% (95% CI 19.92 - 24.68). The OR for SIBO among IBD patients was 9.51 (95% CI 3.39 - 26.68) and significant in both ulcerative colitis (OR = 7.96; 95% CI 1.66 - 38.35) and Crohn’s (OR = 10.86; 95% CI 2.76 - 42.69). The results of the study support the idea that IBD does indeed place patients at higher risk for bacterial overgrowth.

The link between PPI use and SIBO has proven to be controversial in the past as initial studies failed to show a direct correlation between their use and increased susceptibility for bacterial overgrowth. However, subsequent studies have confirmed an association between PPIs and SIBO [9]. A recent retrospective study (n = 1,263 duodenal aspirates) showed that among patients with positive culture results, PPI usage was much higher when compared to those with negative culture results (52.6% vs 30.2%, respectively) [10]. Moreover, a 2018 meta-analysis reviewing 19 studies (N = 7,055) confirmed a higher risk of small intestinal bacterial overgrowth following extended use of PPIs (OR 1.7; 95% CI 1.2 - 2.4) [9].

Structural abnormalities involving the ileocecal valve have also been cited as a potential risk factor for SIBO. The proposed mechanism involves abnormal or inappropriate reflux of colonic microbiota into the ileal portion of the small intestine [11]. A study aimed to assess the relationship between ileocecal valve pressures and SIBO concluded that low ileocecal valve pressures do predispose patients to bacterial overgrowth in the small intestine, with positive results observed in 15 out of 23 subjects (65.2%), as determined by positive lactulose breath testing (LBT) [12]. Ileocecal junction pressures were significantly higher in LBT-negative subjects compared to LBT-positive subjects (79.9% vs. 45.1%, respectively; p < 0.01). Despite this data, additional large-scale studies are needed to further elucidate the relationship between ileocecal valve dysfunction and SIBO.

**Table 1:** Risk Factors for Small Intestinal Bacterial Overgrowth PPI: proton pump inhibitor

Structural Abnormalities	Motility Abnormalities	Biochemical Abnormalities
Small bowel diverticula	Medications (opiates, anticholinergics)	Chronic pancreatitis
Blind intestinal loops	Gastroparesis	Hypochlorhydria (e.g., PPI use, atrophic gastritis)
Adhesions, strictures	Connective tissue disease (e.g., scleroderma)	Common variable immunodeficiency
Ileocecal valve impairment		

Diagnostic evaluation

There are multiple testing modalities available to screen for SIBO; however, each one comes with its own limitations and controversy. The current gold standard for diagnosis remains a quantitative culture of aspirated small bowel fluid. However, the high cost of the procedure, combined with its invasive nature, has made it less than ideal for many patients. Furthermore, limitations of the procedure, including varying bacterial concentrations along with the small bowel and possible contamination by oropharyngeal flora, make it impractical for routine clinical use. Also, it is important to note that a high percentage of the bacteria colonizing the gut cannot be cultured and that patchy distribution of bacteria along with the small bowel that prevents accurate quantification of bacterial overgrowth [13-15].

Breath tests are simple, non-invasive, patient-friendly methods for diagnosing bacterial overgrowth. The practical nature of the procedure and low cost have made it the go-to diagnostic tool in clinical practice. The diagnostic role of hydrogen breath tests largely depends on the type of substrate used. For example, lactose hydrogen breath tests are useful in cases of carbohydrate malabsorption, while lactulose and glucose hydrogen breath tests are useful for diagnosing bacterial overgrowth. In patients with carbohydrate malabsorption, the colonic gut flora produces hydrogen and methane gases from the ingested substrates; in patients with SIBO, the small bowel bacteria produce these same gases. The majority of the gases produced are rapidly eliminated with passing flatus. However, about 20% of the gases are absorbed by the lung and then exhaled, which allows for quantitative measurement during breath testing. Contrary to prior studies, where methane level measurement did not increase the yield of hydrogen breath testing, recent data suggest checking methane levels does increase the diagnostic yield of hydrogen breath testing and should be used for diagnostic purposes [3, 16].

Glucose hydrogen breath testing (GHBT) has been shown to be more specific but less sensitive, yielding a higher rate of false-negatives and a lower rate of false-positives. The specificity and sensitivity of the GHBT range anywhere from 78% - 97% and 15.7% - 62%, respectively. In contrast, lactulose testing is more sensitive but less specific, with a reported sensitivity of 31% - 68% and specificity of 65% - 97.9%. A recently published North American consensus on hydrogen breath testing has characterized cutoff values for abnormal breath testing, a useful reference for those unsure of how to interpret diagnostic results [3]. The guidelines state that a rise in the hydrogen of ≥ 20 ppm (parts per million) within 90 minutes during glucose or lactulose breath testing should be considered as a positive result. Also, a rise in methane levels by ≥ 10 ppm should be considered methane-positive. The same report listed consensus doses for glucose, lactulose, lactose, and fructose breath tests as 75, 10, 25, and 25 g, respectively.

**Figure 1:**
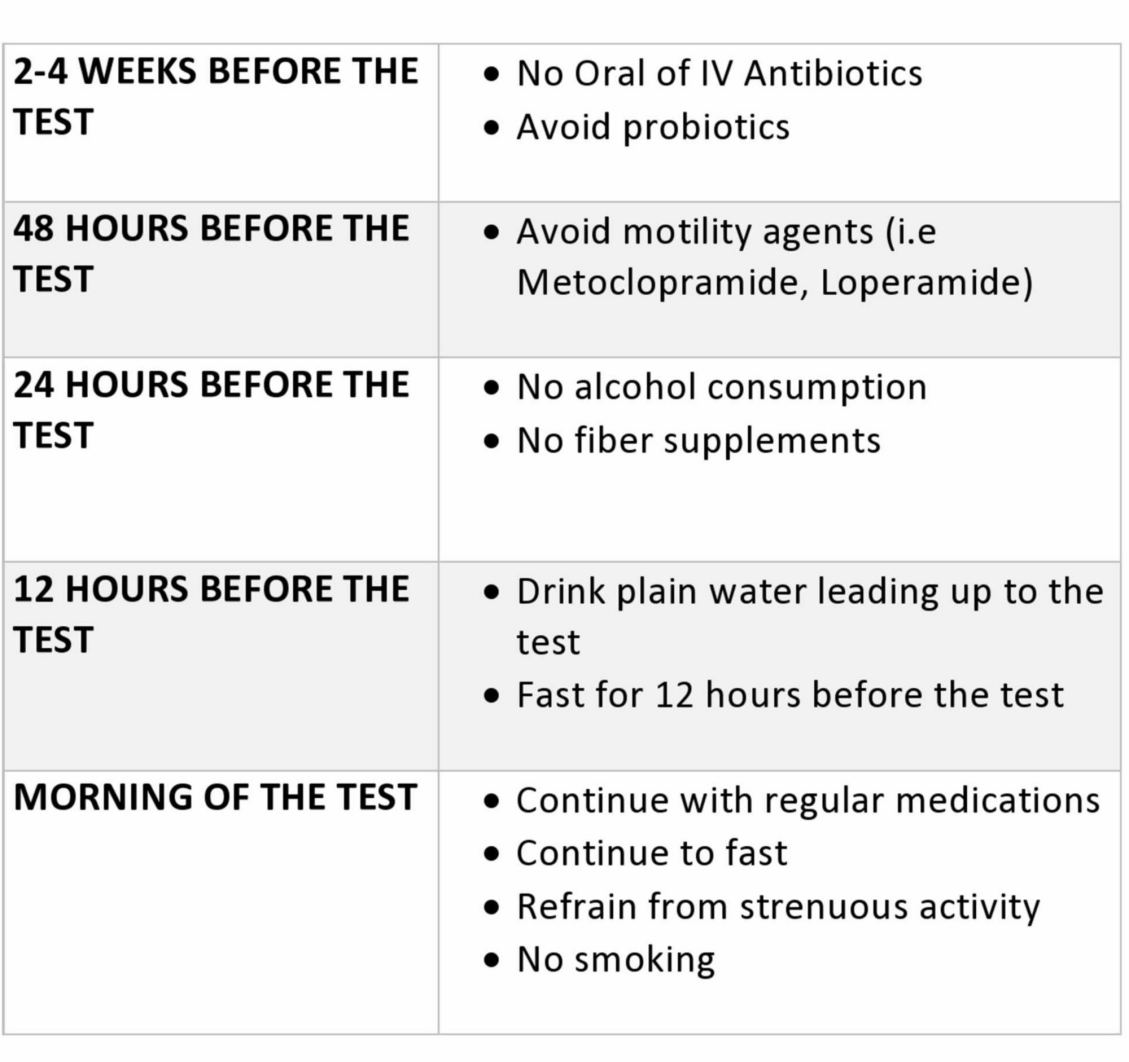
Breath test preparation IV: intravenous

Pre-testing preparation plays a vital role in obtaining accurate diagnostic data (Figure 1). Guidelines recommend stopping antibiotics prior to testing as their use has been linked with altered hydrogen and methane composition of the exhaled breath [3, 17]. Despite the lack of clear-cut data, a four-week gap between antibiotic cessation and diagnostic testing is generally recommended [3, 18]. A low fasting level of breath hydrogen is crucial for proper interpretation of breath test results, as hydrogen levels are directly affected by the consumption of fermentable complex carbohydrates [18-19]. Therefore, it is now recommended that patients avoid complex carbohydrates and dairy products the evening before or 24 hours prior to undergoing testing. Additionally, smoking increases exhaled hydrogen concentrations and should be avoided on the day of the testing as suggested by the recently published North American consensus on hydrogen and methane breath testing [3].

Supportive lab data can be used to validate SIBO suspicion in cases where diagnostic modalities were inconclusive. SIBO is classically associated with nutritional deficiencies, with vitamin B12 levels commonly affected due to inhibited absorption and/or competitive bacterial uptake. While there are certain B12-producing bacteria, the majority of gut bacteria are consumers, causing a nutritional deficiency. Folate levels are frequently increased in SIBO as the vitamin is a byproduct of bacterial metabolism [20]. Deficiencies of fat-soluble vitamins (A, D, E, K) as a result of fat malabsorption have also been reported, occasionally presenting with clinically significant implications, ranging from decreased bone density, osteoporosis, and neuropathy [21].

Despite the popularity of hydrogen breath testing, there are certain limitations that make this diagnostic method less than perfect. GHBT is oftentimes falsely negative among those with distal SIBO, as glucose is completely reabsorbed in the proximal small bowel and often times does not reach the site of bacterial overgrowth. Similarly, in patients with fast gut transit, hydrogen breath tests often yield false-positives due to early substrate delivery to the colon, increasing the chance of a false-positive result.

To overcome the shortcomings of currently available testing modalities, new diagnostic techniques are now being explored. Culture-independent approaches have proved to be successful in the discovery of new bacterial species. Recently developed molecular techniques allow for the identification of different bacterial species based on the sequences of their 16S ribosomal RNA (16S rRNA) genes, present in all microbes [21]. Metagenomics, defined as the analysis of genetic material, can be used to reconstruct bacterial genomes and study the gut microbiome diversity and dysbiosis. First described in 1998 by Handelsman and Rodon, metagenomics aims to catalog genes by the random sequencing of all DNA extracted from the sample [22]. Moreover, metagenomics can identify microbial pathways, antibiotic resistance genes, and determine interactions and co-evolution between microbiota and host [23].

As a diagnostic tool, metagenomics remains in infancy stages. Therefore, combining other microbiome approaches, including cultivation methods, with a study of metagenomics allows for more accurate and convincing findings. Recent studies have successfully used this combination to identify new bacterial strains. The human gut is not only inhabited by bacteria but also by eukaryote and viruses. To date, there have been successful studies carried out on eukaryote and viruses using the metagenomics approach, making it a promising tool in the future investigation of the human gut microbiome [23].

Conventional therapy

Systemic Antibiotics

Given the limitations of current diagnostic techniques, clinicians often initiate empiric therapy as a diagnostic tool in those with a high level of suspicion for SIBO. In this context, the improvement of symptoms following a trial of antibiotics would lean providers towards making the diagnosis. However, this strategy in itself can be problematic as it exposes patients to risks of antibiotic therapy, including the development of antibiotic-resistant organisms and infections (i.e., *Clostridium difficile* colitis).

Traditionally, the go-to antibiotics for treatment of SIBO consisted of tetracyclines, fluoroquinolones, and co-trimoxazole. However, rifaximin has emerged as the preferred agent among clinicians for SIBO management. Rifaximin is a nonabsorbable antibiotic which acts against Gram-positive and Gram-negative aerobic and anaerobic bacteria. The preferred use of rifaximin stems from its reduced toxicity profile and its utility in irritable bowel syndrome, a diagnosis with significant clinical overlap with SIBO. Furthermore, data shows that rifaximin can act as a “eubotic” agent by preserving colonic flora while increasing the relative abundance of lactobacilli and bifidobacteria in the gut [24]. The eradication rate of SIBO also seems to be dose-related. A previously conducted study reported a dose-dependent eradication rate where higher doses of rifaximin were associated with a higher eradication rate [25]. In a recent meta-analysis aimed at investigating the effectiveness of rifaximin in bacterial overgrowth, the efficacy of rifaximin in eradicating SIBO was 64% as compared to 41% with other systemic antibiotics, including tetracyclines and metronidazole [26]. Another meta-analysis looking at eight studies showed that the effectiveness of rifaximin in the normalization rate of breath testing was 49.5% [27].

Alternative and nutritional therapy

A variety of alternative therapies have been proposed over recent years, many of which have originated outside the medical community. Despite the lack of supporting data, alternative therapy can represent a realistic option for those unresponsive to traditional treatment methods.

Probiotics

Probiotics are live microorganisms, which can alleviate the symptoms of SIBO when administered in sufficient quantities. Probiotics act by multiple mechanisms, including modulation of gut microbiota, sustaining the integrity of intestinal epithelium, upregulating anti-inflammatory cytokines and growth factors, production of short-chain and branched-chain fatty acids, as well as interacting with the brain-gut axis by regulating endocrine and neurologic functions [28]. A recent meta-analysis suggested that probiotics are effective in reducing the bacterial burden in SIBO patients and alleviating their symptoms [29]. Furthermore, probiotics may enhance the effectiveness of antibiotics as demonstrated in a recent study where patients treated with rifaximin along with probiotics (*Lactobacillus casei)* had greater improvement in their symptoms with dual therapy as opposed to antibiotics alone [30]. In contrast, a recent study has also shown that probiotics may provoke symptoms among SIBO patients including gas, bloating, and brain fogginess [31]. In the study, probiotic cessation, and a course of antibiotics, resolved brain fogginess while improving other gastrointestinal symptoms (p = 0.005) in 23/30 subjects (77%). This suggests that not all probiotics are of equal efficacy and should be used with caution in patients with SIBO. A 2018 study aimed at assessing how recent probiotic use effects breath testing yielded interesting results that have some questioning the role of probiotics in SIBO management (Mitten E, Goldin A: S660: Recent probiotic use is independently associated with methane-positive breath test for small intestinal bacterial overgrowth. Presented at the 2018 American College of Gastroenterology Annual Scientific Mtg. and Postgraduate Course, October 5-10, 2018, Philadelphia, PA). The study showed that probiotic use within one month was independently associated with increased methane positive LBT in patients presenting with suspected SIBO symptoms. Probiotic users were significantly more likely to have positive LBT compared to non-users (93.6% vs 65.7%, p = 0.003). More specifically, those individuals with recent probiotic use were more likely to have methane-positive LBT but not hydrogen-positive LBT. These findings suggest that probiotic use can predispose to overgrowth of methanogenic bacteria. The use of probiotics can potentially increase the risk for methane predominant variant of SIBO which has been associated with constipation-predominant symptoms. The lack of clear consensus regarding probiotic use suggests that additional large scale studies are needed to better understand the effects of probiotics on SIBO risk.

Herbal Supplements

Herbal supplements marketed for SIBO relief are widely available and have become increasingly more popular as many are turning away from traditional pharmacological therapy in search of alternative and cheaper methods. A recent study has shown that herbal supplementation can be as effective as rifaximin as measured by a negative follow-up breath test. The study (n = 251) looked at patients who tested positive for SIBO following lactulose breath testing (LBT). In the study, subjects with newly diagnosed SIBO were given two treatment choices; either two 200 mg rifaximin tablets three times daily (TID) or two capsules twice daily of the following commercial herbal preparations: Dysbiocide® and FC Cidal™ (Biotics Research Laboratories, Rosenberg, TX) or Candibactin-AR® and Candibactin-BR® (Metagenics, Inc, Aliso Viejo, California) for four consecutive weeks immediately followed by a repeat LBT. Results showed that 17/36 subjects on herbal supplementation (46%) had a negative follow-up LBT compared to 23/67 (34%) of rifaximin users. The odds ratio of having a negative LBT after taking herbal therapy as compared to rifaximin was 1.85 (CI = 0.77 - 4.41, P = .17) once adjusted for gender, age, and SIBO risk factors. The same study concluded that herbal therapy has similar efficacy as triple antibiotic therapy for SIBO rescue therapy for rifaximin non-responders [32]. However, it’s important to note data regarding herbal supplements for SIBO is extremely limited and products currently available differ significantly in composition and quality.

Diet

Dietary manipulation can be beneficial for relieving symptoms of SIBO including bloating, flatulence, and abdominal pain. In patients with SIBO, gut bacteria ferment carbohydrates, such as fructose, lactose, oligosaccharides, disaccharides, and monosaccharides, resulting in gas formation and the aforementioned symptoms. The low FODMAP (fermentable oligosaccharides, disaccharides, monosaccharides, and polyols) diet is probably the best-known diet for SIBO; however, most data on its effectiveness is based on IBS, which has significant clinical overlap with SIBO. FODMAP represents a list of sugars that can be fermented in the gut. Select bacteria inhabiting the small bowel thrive by consuming FODMAPS, therefore, limiting their number deprives bacteria of the much-needed nutrition required for growth and proliferation. Although effective, FODMAP diets can be complex and hard to adhere to without expert guidance. Most patients benefit from professional expertise on the different phases of the diet, trigger identification, slow elimination of foods, and their reintroduction into the daily diet. Furthermore, data shows that diets rich in complex carbohydrates may favor the proliferation of less pathogenic bacteria when compared to diets rich in fat or protein [33]. Vegan and vegetarian diets rich in fiber have proved to be effective for many with SIBO symptoms. These diets increase the production of short-chain fatty acids, while simultaneously inhibiting potentially invasive bacteria, such as *Escherichia coli* and other members of *Enterobacteriaceae* species [34].

A 2019 study aimed at analyzing nutrition patterns in patients with treatment-resistant SIBO, showed that treatment-resistant subjects had higher consumption of buckwheat compared to treatment responsive subjects (0.41 ± 0.47 vs 0.14 ± 0.35, p < 0.001), poultry meat (0.80 ± 0.64 vs 0.54 ± 0.62, p = 0.01), millet (0.036 ± 0.11 vs 0.007 ± 0.021, p = 0.047), and butter (0.54 ± 0.24 vs 0.39 ± 0.22, p < 0.01) [35]. The diet of patients with treatment-resistant SIBO was also significantly lower in mono- and disaccharides (75.2 ± 32.7 vs 95.5 ± 41.5 g/day; p = 0.015). This data can be used to guide dietary plans for the maintenance of SIBO therapy and prevention of symptom relapse.

An elemental diet consisting of predigested micronutrients has also been suggested as an option to potentially relieve symptoms of SIBO. The proposed benefit of the diet stems from the high amount of predigested micronutrients that are mostly absorbed within the proximal small bowel, thus limiting the delivery of nutrients to the distal portion of the small bowel. A retrospective study examined the potential benefit of elemental diet among patients with SIBO. In the study (n = 124) patients were treated with an elemental diet for a period of 14 days. The overall symptomatic response rate among this cohort was 85%, as indicated by normalization of breath tests [36]. Despite promising data, such a diet may not be sustainable for many due to high cost, especially for those without prescription or insurance coverage. While dietary modification represents a short-term and therapeutic maneuver, the idea that it can treat bacterial overgrowth long term should be avoided, as often times the underlying risk factors for SIBO remain.

Treatment failure

**Figure 2:**
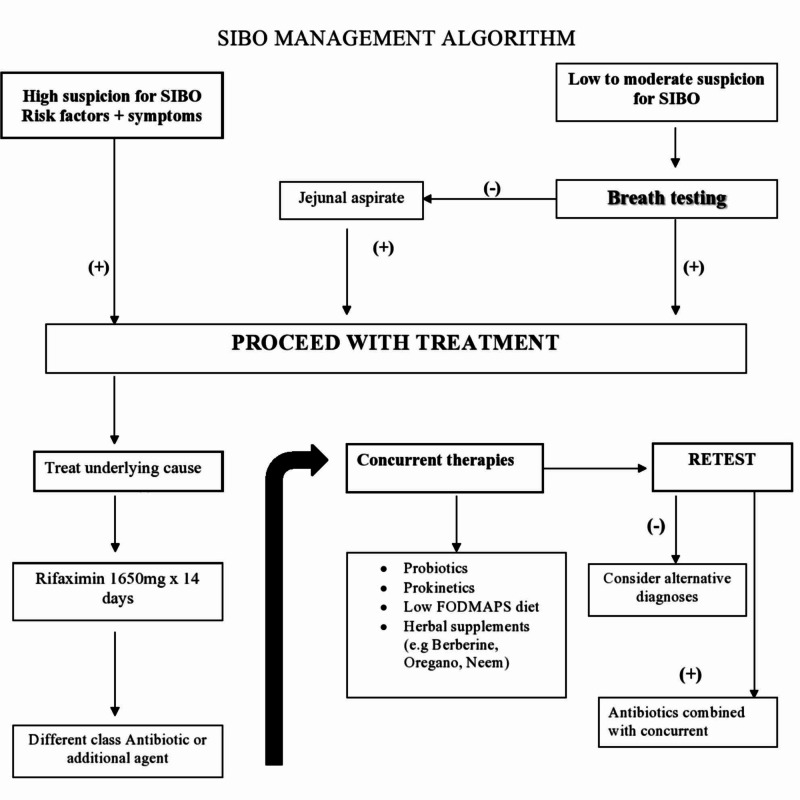
SIBO management algorithm FODSMAPS: fermentable oligosaccharides, disaccharides, monosaccharides, and polyols; SIBO: small intestinal bacterial overgrowth

Despite the proven efficacy of antibiotics for symptom relief, approximately 40% of patients with SIBO-like symptoms may not experience the resolution of their symptoms with antibiotic therapy. Those who fail standard therapy should undergo evaluation for other overlapping diagnoses (Figure 2). Among those, disaccharide deficiency or food intolerance are the most commonly diagnosed conditions in the outpatient setting. Patients with dual conditions, such as SIBO and lactose intolerance, might only experience partial relief of their symptoms with antibiotic therapy and will require lactose-free diets long-term. As such, a thorough assessment of symptoms and appropriate diagnostic testing is required to rule out other conditions in those with suboptimal response to antibiotic therapy. 

## Conclusions

SIBO remains a widely prevalent diagnosis in tertiary referral gastroenterology practice. While there has been significant progress made in our understanding of the condition, efforts to fully unravel this complex diagnosis remain hampered by the limitations of currently available diagnostic tools. Although a perfect diagnostic test for SIBO is lacking, currently available breath testing has proven to be a safe and preferred method in clinical practice. With the lack of clear-cut criteria and diagnostic tools, it is harder to prove the diagnosis of SIBO when it is suspected. This may soon change, however, as the application of molecular techniques to the study of the small intestinal microbiome, coupled with innovative sampling techniques, may soon enable clinicians to truly define the spectrum of SIBO. Additional studies are needed to further characterize contributing pathophysiological mechanisms in SIBO and to investigate optimal treatment for this challenging patient population. While SIBO continues to be a controversial diagnosis, in the era of booming microbiome research, gastroenterologists and other clinicians will surely become increasingly aware of SIBO in the general population, enabling them to provide more effective treatment.
