# Correction: Small Intestinal Bacterial Overgrowth: Comprehensive Review of Diagnosis, Prevention, and Treatment Methods

**DOI:** 10.7759/cureus.c227

**Published:** 2025-07-03

**Authors:** Ted George O Achufusi, Anuj Sharma, Ernesto A Zamora, Divey Manocha

**Affiliations:** 1 Internal Medicine, State University of New York Upstate Medical University, Syracuse, USA; 2 Gastroenterology, State University of New York Upstate Medical University, Syracuse, USA

This article has been corrected to fix the following typo: "a rise in the hydrogen of ≥ 20 ppm (parts per million) **after** 90 minutes during glucose or lactulose breath testing should be considered as a positive result." The bolded word "after" has been replaced with "within" as follows: "a rise in the hydrogen of ≥ 20 ppm (parts per million) within 90 minutes during glucose or lactulose breath testing should be considered as a positive result."

